# Weight Bias Internalization: The Maladaptive Effects of Moral Condemnation on Intrinsic Motivation

**DOI:** 10.3389/fpsyg.2018.01836

**Published:** 2018-09-27

**Authors:** Susanne Täuber, Nicolay Gausel, Stuart W. Flint

**Affiliations:** ^1^Department of Human Resource Management and Organizational Behavior, University of Groningen, Groningen, Netherlands; ^2^Department of Psychosocial Health, University of Agder, Kristiansand, Norway; ^3^Carnegie School of Sport, Leeds Beckett University, Leeds, United Kingdom

**Keywords:** weight stigma, moralization, incompetence, weight bias internalization, motivation, maladaptive and adaptive functioning

## Abstract

Weight stigma typically focuses on suggestions that people with overweight and obesity are incompetent and immoral. Integrating so far unconnected lines of research, the current research presents two studies that examine the motivational relevance of these aspects of weight stigma. Specifically, we tested the proposition that people with overweight and obesity respond differently to the public viewing them as incompetent compared to immoral, as these aspects of weight stigma differ in reparability. We expect that threats to competence are more acceptable and thus related to a constructive response that is more effective in losing weight in the long-run. By contrast, we propose that threats to morality elicit an acute urge to defend one’s moral image, thereby prompting responses that are more visible to the social environment, but potentially less effective for losing weight. Study 1 experimentally compared exposure to weight stigma focused on morality vs. weight stigma focused on competence in a sample of adults with overweight and obesity (*N* = 122; *M*_BMI_ = 31.89, *SD*_BMI_ = 4.39). We found that when exposed to weight stigma focused on morality, people with overweight and obesity respond by defending their moral social-image but that this is less effective for encouraging weight loss, while exposure to weight stigma focused on competence led to an increased likelihood of engagement in weight loss behaviors. Complementing and extending the findings, Study 2 (*N* = 348, *M*_BMI_ = 26.78, *SD*_BMI_ = 6.78) tested the notion that internalized weight bias predominantly revolves around moral concerns, and thus will lead to less self-determined behavioral regulation. We found strong support for the moral core of weight bias internalization. In line with our predictions, greater weight bias internalization was associated less self-determined and more other-determined regulation of dieting and exercising. This suggests that weight bias internalization operates as a facilitator of maladaptive behavioral regulation following weight stigma, contributing to lower psychological functioning and well-being of people with overweight and obesity. The current research presents novel findings about the underlying mechanisms of weight stigma and weight bias internalization and identifies strategies to avoid maladaptive and facilitate adaptive health behaviors.

## Introduction

Weight has become a pervasive topic that is typically framed in moral terms such as in policy discourse, media portrayal, and public settings (e.g., Rozin, 1999; Townend, 2009; Flint et al., 2016b). For many years, scholars and politicians alike have offered opinions and debated the morality of public health as a means of increasing motivation in those targeted to engage in “healthier behavior” (Conrad, 1994; Bossy, 2010; Brown, 2013). However, rather than a decrease in the prevalence of overweight and obesity, which one would expect if the above strategy was successful, there has been a steady increase in prevalence rates (World Health Organisation [WHO], 2017). In addition, previous literature (e.g., Jackson et al., 2015) has reported that weight stigma leads to maladaptive health behaviors such as unhealthy eating and avoidance of exercise settings. Moreover, people with overweight and obesity of all ages and backgrounds report experiences of weight stigma and discrimination (e.g., Puhl and Luedicke, 2012; Flint et al., 2015). Weight stigma typically refers to depictions of people with overweight and obesity as lacking willpower, being lazy, unintelligent, and gluttonous (Puhl and Brownell, 2006). In all domains of life and work, stigma has been associated with discrimination (Link and Phelan, 2001). For instance, previous research (e.g., Roehling et al., 2007; Bartels and Nordstrom, 2013; Flint et al., 2016a) has reported that people with overweight and obesity applying for employment are assessed as less suitable and as lacking leadership qualities compared to applicants without obesity. Two fundamental elements of weight stigma are perceptions that people with overweight and obesity lack competence (i.e., unintelligent) and are immoral (i.e., gluttonous). In this regard, the – unsuccessful – moralized framing of overweight in political and public discourse (Rozin, 1999; Townend, 2009; Flint et al., 2016b) reflects an emphasis on the moral aspect of overweight. Taken together, the emerging picture is one where ongoing and pervasive weight moralization fails to achieve the desired changes in weight status. On the contrary, weight moralization appears to demotivate and trigger maladaptive responses to weight stigma. The main aim of this research was to advance scholarly understanding of the mechanisms underlying maladaptive behavioral responses to weight stigma (e.g., Haines et al., 2006). To achieve this aim, we integrate previously unconnected lines of research on moral motivation with insights into self-defense, self-improvement, and weight bias internalization. Based on this integration and the findings flowing from it, we offer strategies to support psychological functioning and well-being of people with overweight and obesity.

### Adaptive and Maladaptive Responses to Weight Stigma

In conceptualizing adaptive and maladaptive responses to weight stigma, we build on Self-Determination Theory (SDT; Deci and Ryan, 1985). SDT offers a fruitful theoretical framework to approach eating pathologies (e.g., Pelletier et al., 2004; Pelletier and Dion, 2007) and exercise behavior (Markland and Tobin, 2004). SDT is based on the premise that types of human motivation predict outcomes related to performance, relationships, and wellbeing (Deci and Ryan, 2008). These types of motivation reflect the extent to which the desire to perform a behavior is rooted in the person themselves (autonomous motivation) vs. in others (controlled motivation). On a continuum, intrinsic motivation reflects completely autonomous behavioral regulation, while external motivation reflects completely controlled behavioral motivation. Amotivation stands for a lack of motivation and is thus not associated with behavioral regulation (Ryan and Deci, 2000). Importantly, when autonomously motivated, people experience volition and self-endorse their actions (Deci and Ryan, 2008). By contrast, when under controlled motivation, people experience “pressure to think, feel, or behave in particular ways” (Deci and Ryan, 2008, p. 182). Unsurprisingly, autonomous and controlled motivation lead to vastly different outcomes, with autonomous motivation associated with greater psychological health, persistence, and greater adherence to healthy behaviors than controlled motivation (Deci and Ryan, 2008). Extant research using SDT as a theoretical framework has demonstrated the benefits of autonomous motivation in the context of weight loss. For instance, Pelletier and Dion (2007) found that women with autonomous motivation were more likely to eat healthy and less likely to eat unhealthily. Similarly, Williams et al. (1996) observed more regular attendance and greater weight loss in weight loss program attendees’ who reported greater autonomous motivation. Thus, in stimulating lasting efforts and intrinsic commitment to weight loss, practitioners should attempt to instill a sense of autonomous motivation in clients as this facilitates adaptive strategies to weight loss. On the other hand, practitioners should avoid instilling a sense of controlled motivation in clients, as this appears to be associated with less adaptive strategies to weight loss. The question for policy makers and practitioners alike is, how can “the right kind” of motivation be achieved? To answer this question, we examine the motivational relevance of morality- and competence-related aspects of weight stigma.

### The Motivational Relevance of Threats to Morality

The observation that moralization fails to achieve the desired changes in people’s health outcomes, aligns with research that challenges the effectiveness of moralized persuasion. Specifically, Täuber and van Zomeren (2012, 2013) and Täuber et al. (2015) demonstrated that framing shortcomings as related to people’s morality is likely to result in a refusal to engage in the desired behavior. By contrast, framing the same shortcoming as related to people’s competence motivates people to engage in the desired behavior. This effect has been shown in diverse contexts such as climate change (Täuber and van Zomeren, 2013; Täuber et al., 2015), poverty reduction (Täuber and van Zomeren, 2012), and immigration politics (Täuber and van Zomeren, 2013). The underlying reason for the observed asymmetric impact of competence and morality on motivation is the primacy of morality in impression formation (e.g., Fiske et al., 2007; Täuber and van Zomeren, 2012). While both competence and morality are fundamental dimensions in social judgment, morality is more important (e.g., Wojciszke, 1994, 2005; Leach et al., 2007) both for the understanding of who one is as a person (Gausel and Leach, 2011) and for the impression that others have of oneself (Gausel and Leach, 2011; Ellemers and van den Bos, 2012). Moreover, morality is also key to the maintenance of social bonds (Gausel, 2013) and is viewed as more essential than competence in relation to survival and social inclusion in groups (e.g., Täuber, 2018). This reasoning is supported by research showing that people actively search for cues of immorality in others (e.g., Gantman and Van Bavel, 2014, 2015). Furthermore, cues of immorality are more resistant to counter-information than cues of incompetence (Skowronski and Carlston, 1992). Finally, people make faster and more extreme judgments when morality is concerned (Van Bavel et al., 2012).

Based on the above, it is unsurprising that being perceived as immoral is an aversive experience, and more so than being perceived as incompetent (e.g., Tetlock, 2002; Monin, 2007). Therefore, people try to act morally in the eyes of others (Gausel and Leach, 2011; Gausel, 2013). The idea that emphasizing the moral core of an issue will lead to increased motivation in those targeted to change their behaviors in the desired ways is based on these insights. Unfortunately, an increasing body of research shows that questioning others’ morality is likely to lead to self-protective responses (Monin, 2007; Gausel et al., 2012, 2016; Täuber et al., 2015). Indeed, in a theoretical response to climate change researchers’ plea to frame the urge to act as a moral imperative (Markowitz and Shariff, 2012), Täuber et al. (2015) suggested that because the evaluative relevance of morality is so strong for humans, questioning morality can lead to “defensive overkill” (see also Tetlock et al., 2000).

Thus, when people feel that their moral image is threatened, they may not simply refuse to show the desired change in behavior, but they might disengage from the behavior altogether. The “defensive overkill” response to moral threats might be reflected in maladaptive responses to weight stigma such as binge eating (e.g., Duarte et al., 2014). Indeed, two fundamental elements of weight stigma are perceptions that people with overweight and obesity lack competence (i.e., unintelligent) and are immoral (i.e., gluttonous). In this regard, the – unsuccessful – moralized framing of overweight in political and public discourse (Rozin, 1999; Townend, 2009; Flint et al., 2016b) reflects an emphasis on the moral aspect of overweight and might thus be partly responsible for maladaptive responses to weight stigma. In sum, while moral framing is often used with the intention to intrinsically motivate others to show a desired behavior, moralization will likely achieve the opposite effect, namely disengagement and withdrawal from the behavior. Together, based on this line of inquiry we expect that emphasizing the moral elements inherent to weight stigma will be demotivating.

### Shame, Self-improvement, and Self-defense

Another stream of research is focused on people’s reactions to failure. Public discourse typically depicts people with overweight and obesity as failing to live up to social norms and standards (Duarte et al., 2015; Täuber, 2018). While a conception of people with overweight and obesity as failures does not reflect the authors’ view, we believe that recent research into how people respond to failure might be valuable in understanding responses to weight stigma. This reasoning is based on the notion that, reflecting public opinion, people with overweight and obesity likely perceive themselves as having failed with respect to their weight status (Durso and Latner, 2008). In recent years, research concerning motivational and behavioral responses to failure has aimed to explain why failure leads to self-improvement in some situations while in others leads to self-defensive withdrawal (Gausel and Leach, 2011; Gausel et al., 2012, 2016; Gausel, 2013). Specifically, in their social psychological model, Gausel and Leach (2011) argue that after a self-relevant failure people tend to appraise this failure in two main ways: first, by appraising how the failure affects one’s understanding of oneself (i.e., one’s self-image); and second, by appraising how the failure affects what others think of oneself (i.e., one’s social-image). The feeling of shame is a self-critical feeling (Tangney and Dearing, 2002) that is likely to surface when the self has been associated with a failure (Gausel and Leach, 2011) or when the threat to the self is deemed acceptable (Tetlock, 2002; Monin, 2007; Täuber et al., 2015). Indeed, Leach and Cidam (2015) conducted a meta-analysis to examine the situations in which shame will lead to more constructive approaches (i.e., stimulating self-improvement) and in which situations it will lead to less constructive (i.e., avoidance, withdrawal) behavioral responses. These authors found strong support for the suggestion that self-improvement results from failures that are considered repairable, while self-defense results from failures that are perceived to be less repairable (Leach and Cidam, 2015).

These insights align with research exploring shame in a functionalist perspective (de Hooge et al., 2010; Gausel and Leach, 2011), suggesting that the primary function of shame aims at motivating people to restore a positive self-image. This motivation, however, is moderated by people’s perception of how repairable a failure that leads to shame is (Leach and Cidam, 2015). Since incompetence is more repairable than global immorality (e.g., Skowronski and Carlston, 1992), we expect that when weight stigma focuses on incompetence, people with overweight and obesity will experience feelings of repairable shame for the self-related failure to be competent (e.g., de Hooge et al., 2010). The difference in repairability closely resembles the difference between traits and states: Morality is assumed to reflect people’s true self and inner character and is therefore perceived as stable and resistant to change (Aristotle, 1985; Tangney and Dearing, 2002; Leach et al., 2007; Gausel and Leach, 2011). Competence, on the other hand, is seen as reflecting people’s abilities, which are assumed to be malleable and therefore possible to change through practice and training (Cole, 1991; Harter, 1992). Thus, even though being depicted as incompetent is unpleasant, it is more likely to promote reformatory responses (for a discussion, see Gausel and Leach, 2011; Leach and Cidam, 2015) than being depicted as immoral. It thus seems plausible to assume that people with overweight and obesity who feel shame will be motivated to lose weight for shame-related internal reasons meant for self-change (Gausel and Brown, 2012; Lickel et al., 2014).^1^ On the other hand, overweight and obesity are pervasively moralized in public discourse (e.g., Rozin, 1999; Townend, 2009; Flint et al., 2016b). Morality is strongly associated with ascriptions of control and leads to an often-incorrect assumption that an outcome is representative of effort (Täuber, 2018). This means that a core public assumption regarding overweight and obesity is that it reflects a lack of effort in the regulation of eating and exercising. In addition, being seen as immoral is less repairable than being seen as incompetent (Skowronski and Carlston, 1992). This aligns with prior research suggesting that being immoral is perceived as a much more global flaw than being incompetent (Gausel and Leach, 2011) and therefore much more problematic than incompetence. Consistent with this, de Hooge et al. (2010) showed that shortcomings in the competence domain often lead people to prove their competence. We thus propose that when weight stigma predominantly suggests that people with overweight and obesity are globally immoral, being overweight will elicit a constant fear of being morally condemned by the public, especially given that overweight is a visible stigma (Crocker and Major, 1989; Weiner, 1995).

A direct way to minimize anticipated condemnation is to engage in social appeasement or pleasing strategies that might better ones standing with others (Gausel and Leach, 2011; Gausel, 2013). Such strategies aim to communicate to others that one is morally exemplary, thereby seeking to contrast the (anticipated or actual) public condemnation of people with overweight and obesity as immoral. These can involve relatively ineffective, low-cost behavior such as promising to search for information on healthy lifestyles, but they might also involve complete disengagement from the topic, as suggested by the “defensive overkill” sometimes prompted by threats to morality (see Täuber et al., 2015). As noted above, research showing that weight stigma leads to binge eating (e.g., Haines et al., 2006; Duarte et al., 2014) and refusal to diet (Puhl et al., 2007) might provide a tentative reflection of such “defensive overkill” in the weight domain. Observing maladaptive or relatively less effective behavioral responses, when weight stigma suggests that people with overweight and obesity are immoral, thus likely reflects a functional approach to managing an extremely adverse threat to one’s moral image. We suggest that in such situations, to deal with the threat, people will prefer more visible strategies that can be implemented quickly (such as getting brochures about healthy eating) over less visible strategies that require more time (such as losing weight). Visibility in this context refers to how easily observable a behavior is to the social environment. While dieting might be more effective in the long-run when trying to lose weight, it is less easily observable to the social environment than getting and reading brochures about healthy eating. In this sense, there might be an important trade-off, where the effectiveness of signaling to the social environment that one is working at losing weight comes at the cost of the effectiveness of the method chosen to lose weight.

### Weight Bias Internalization

There are strong reasons to believe that weight bias reflects a moral stance on weight. For instance, in their development of the original weight bias internalization scale, Durso and Latner (2008) contend that the main difference between anti-fat attitudes and internalization of weight bias is the type of attribution made. In particular, Durso and Latner (2008) suggest that because internalization of weight bias involves making harmful assumptions about the self rather than about the other, it potentially harms those who internalizes weight bias. However, the beliefs underlying self-directed bias will parallel the beliefs underlying other-directed bias. Thus, while we are not aware of explicit attempts to associate weight bias internalization with morality, research into other-directed stigma converges in the notion that controllability beliefs are a crucial determinant of stigma (e.g., Weiner et al., 1988; Weiner, 1995). This holds for all stigma but has also been demonstrated for obesity (Tiggemann and Anesbury, 2000). Importantly, controllability and responsibility attributions are paramount to seeing an issue as moral. If an outcome is not under people’s control, failing to achieve the outcome will not lead to others attributing this failing to a lack of morality (Weiner, 1995). Thus, based on the established link of controllability attributions with anti-fat attitudes (e.g., Weiner et al., 1988; Crandall, 1994; Crandall et al., 2001), we suggest that internalized weight bias is also based on attributions of controllability, and thus is inherently associated with morality. Following this reasoning, we predict that, besides own BMI, which has been shown to be associated with weight bias internalization in prior research (for a systematic review, see Pearl and Puhl, 2018), a moral focus on weight stigma rather than a focus on competence, and fear of condemnation will predict weight bias internalization. To the extent that internalized weight bias reflects morality-related concerns more than competence-related concerns, our review above suggests that it should be associated with more controlled and less autonomous motivation. In particular, people with high internalized weight bias should report less self-determined motivation and more other-determined motivation, reflecting their concerns about their social image.

### The Present Research

We designed two studies to test the predictions derived from integrating the different lines of research reviewed above. In a sample of adults with overweight and obesity, Study 1 experimentally varied whether the public’s stigmatized view revolved around people with overweight and obesity being immoral vs. incompetent. We measured respondents’ shame (reflecting self-image concerns), their fear of condemnation (reflecting social image concerns), as well as their preference for more or less visible responses to weight-stigma. Our theoretical integration suggests that the greater reparability of competence-related weight stigma should be reflected in a preference for less visible responses in people with overweight and obesity that require more time, such as losing weight (Hypothesis 1a). This effect should be mediated by experienced shame, thus by concerns about self-image (Hypothesis 1b). On the other hand, the lower reparability of morality-related weight-stigma should be reflected in a preference for more visible responses in people with overweight and obesity that can be implemented quickly, such as getting brochures about healthy eating (Hypothesis 2a). This effect should be mediated by fear of condemnation, thus by concerns about social image (Hypothesis 2b).

In Study 2, we conducted a survey focused on weight bias internalization sampling adults across the weight spectrum. This study testing the suggestion that internalized weight bias predominantly reflects threats to morality (Hypothesis 3). Further, we also measured motivation with specific scales building on SDT (Ryan and Deci, 2000; Deci and Ryan, 2008), to test the notion that internalized weight bias operates as a powerful antecedent of self-determined vs. other-determined behavioral regulation. Specifically, we explored the notion that, due to its strong moral connotation, weight bias internalization is related to less self-determined and more other-determined behavioral regulation of dieting and exercising (Hypothesis 4).

## Study 1

### Participants and Design

Respondents were approached through a research assistant’s network. Specifically, a random sample of 4310 people from a Dutch panel on public transport were invited to participate in a questionnaire about health and lifestyle. After providing informed consent, respondents completed the questionnaire. Of the people invited, 1300 started the questionnaire (response rate 30.16%). Respondents were first asked to indicate whether they considered themselves a person with normal weight (1), with a little overweight (2), with overweight (3), or with a lot of overweight (4). Of the sample, 455 (43.1%) identified as persons with normal weight, 352 (33.3%) identified as persons with a little overweight, 212 (20.1%) identified as persons with overweight, and 37 (3.5%) identified as persons with a lot of overweight. Respondents identifying as normal weight or a little overweight were redirected to another study. Respondents identifying as persons with overweight and a lot of overweight (*N* = 249) were forwarded to the present research. Of those initially starting the study, respondents who did not fill in the complete questionnaire (*N* = 36) were not considered for the analyses, leaving a final sample of 213 respondents who self-identified as overweight (111 female, 102 male; *M*_age_ = 58.50, *SD*_age_ = 11.43; *M*_BMI_ = 31.89, *SD*_BMI_ = 4.39).

The study was presented using the online survey tool Qualtrics^TM^, and respondents were randomly assigned to the conditions of a one-factorial between-subjects design with two levels [the public’s view on overweight: immoral (*N* = 111) vs. incompetent (*N* = 101)].

### Measures

Respondents read an article ostensibly published in an online journal about how lifestyle partly affects the rising healthcare costs. Depending on the experimental condition, the article concluded that “In recent years, public opinion is that an unhealthy lifestyle and therefore also people with an unhealthy weight, are immoral/incompetent.” **Supplementary Appendix A** provides a detailed overview over all measures, as well as the manipulations used in Study 1. The public’s view on overweight was measured by four items, two of which tapped into morality (*r* = 0.65, *p* < 0.001), and two into competence (*r* = 0.80, *p* < 0.001). Shame and concern for condemnation were measured with three items each, adapted from Gausel et al. (2012; 2016, α = 0.90 and α = 0.92, respectively). Wanting to improve lifestyle in general was measured with two items (*r* = 0.53, *p* < 0.001). This measure reflected a more visible, but in the long-run less effective, response to weight stigma. Wanting to lose weight was measured with two items (*r* = 0.74, *p* < 0.001). This measure reflected a less visible, but in the long-run more effective, response to weight stigma. In a review of weight loss maintenance and weight regain, Elfhag and Rössner (2005) suggested that an internal motivation to lose weight is important for weight maintenance. Likewise, the importance of motivation to lose weight on overall effectiveness of losing weight has been reported (Silva et al., 2011) and has led to programs being designed that focus on increasing weight loss motivation in patients and attendees (for an example, see West et al., 2011). The desire to lose weight can also be conceived of as an implementation intention, which have been shown to be effective in reducing the intention–behavior gap (Gollwitzer and Sheeran, 2006).

### Results

#### Manipulation Checks

An ANOVA with experimental condition as between-subject factor and weight stigma as the dependent variable revealed a significant effect for weight stigma as immoral, *F*(1,211) = 10.75, *p* = 0.001, η^2^ = 0.05. Respondents reported that the public views people with overweight and obesity as immoral to a significantly greater extent in the immoral weight-stigma condition (*M* = 2.94, *SD* = 1.06) than in the incompetent weight-stigma condition (*M* = 2.49, *SD* = 0.94). Further, a significant effect of weight stigma as incompetent was evident, *F*(1,211) = 24.66, *p* < 0.001, η^2^ = 0.11. Respondents reported that the public views people with overweight and obesity as incompetent to a significantly greater extent in the incompetent weight-stigma condition (*M* = 2.81, *SD* = 1.09) than in the immoral weight-stigma condition (*M* = 2.08, *SD* = 1.03). The manipulation can thus be considered successful.

#### Descriptive Analyses

**Table 1** displays means, standard deviations, and correlations between the measured constructs. First, respondents’ sex and age were uncorrelated with the dependent variables, except for shame: females and younger respondents reported marginally more and significantly more shame, respectively. Respondents’ BMI was strongly associated with shame and concern for condemnation. Shame was highly correlated with concern for condemnation and with both the quicker and more visible response (seeking information) and with the slower and less visible (losing weight). Concern for condemnation was related only to the quicker and more visible response (seeking information) but unrelated to the slower and less visible (losing weight).

**Table 1:** Descriptive statistics and correlations, Study 1.

		*M* (*SD*)	1	2	3	4	5	6	7
Control variables	1. Gender (1 = male, 2 = female)	1.52 (0.50)	1.00						
	2. Age	58.50 (11.43)	–0.11	1.00					
	3. BMI	31.89 (4.39)	0.04	–0.04	1.00				
	4. Condition^a^	0.50 (1.00)	–0.01	–0.01	–0.01	1.00			
DVs	5. Shame	2.35 (1.03)	0.13^+^	–0.18**	0.25***	–0.02	1.00		
	6. Fear of condemnation	2.15 (1.00)	0.09	–0.12	0.23**	–0.04	0.78***	1.00	
	7. Seek information	2.70 (0.76)	–0.01	0.13	0.00	–0.09	0.14*	0.19**	1.00
	8. Lose weight	4.04 (0.62)	0.10	–0.10	0.03	–0.01	0.17*	0.09	0.23**

*^*a*^1, public views overweight people as immoral (*N* = 111), -1, public views overweight people as incompetent (*N* = 101). ^+^*p* = 0.05, ^∗^*p* < 0.01, ^∗∗^*p* < 0.001.*

#### Hypotheses Testing

We predicted that weight stigma focusing on morality leads to a preference for quicker and more visible, but potentially less effective responses, and that this effect is mediated by concern for condemnation, but not for shame. By contrast, we expected that weight stigma focusing on competence leads to a preference for slower and less visible, but potentially more effective responses, and that this effect is mediated by shame, but not by concern for condemnation. We tested these predictions using structural equation modeling.

### Structural Regression Modeling

**FIGURE 1:**
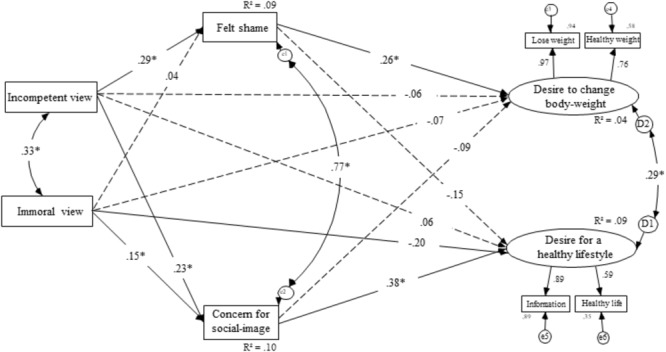
Structural equation model for the effects of feeling morally judged on willingness to improve lifestyle in general through restitution motivation and self-defensive motivation for Study 1.

In line with our hypotheses, we specified a structural regression model using AMOS 23 with maximum-likelihood estimation where the two public views of people with overweight and obesity represented as manifest variables were allowed to predict the two manifest variables of felt shame and the concern for condemnation. Again, this predicted our two main latent variables for this first study; the motivation to change one’s body weight (adaptive behavior aimed at self-betterment) and the motivation for a healthy lifestyle (maladaptive behavior aimed at pleasing others). **Figure 1** displays the model. The structural regression model fit the data very well as indicated by a non-significant chi-square, χ^2^(9) = 4.59, *p* = 0.87 (χ^2^/*df* = 0.51), as well as other fit indices, IFI = 1, CFI = 1, RMSEA = 0.000. As expected, the feeling of shame was significantly predicted by the public view that people with overweight and obesity are incompetent (β = 0.29, *p* < 0.001), but it was not predicted by the view that people with overweight and obesity are immoral (β = 0.04, *p* = 0.61). In contrast the concern for one’s social image was mostly predicted by the public view that people with overweight and obesity are incompetent (β = 0.23, *p* < 0.001), and to a lesser degree; the view that people with overweight and obesity are immoral (β = 0.15, *p* = 0.029). In line with our hypotheses, the feeling of shame was a positive, significant predictor of the desire to change one’s body weight (β = 0.26, *p* = 0.020), and it was a negative, non-significant predictor of the desire for a healthy lifestyle (β = -0.15, *p* = 0.22). Also, in line with our hypotheses, the concern for social image (i.e., the concern for public condemnation) was a positive predictor of a desire for a healthy lifestyle (β = 0.38, *p* = 0.009) and a negative, non-significant predictor of a desire to change one’s body (β = -0.09, *p* = 0.43).

### Discussion

Study 1 provides support for our proposition that weight stigma focusing on immorality facilitates a social threat of condemnation. For people with overweight and obesity, the social threat of condemnation appears to lead to preferences for quickly implementable and visible responses to weight-stigma. We have suggested that preference for such response reflect a functional approach to managing extremely adverse threats to moral social image. Because the threat to moral image is so unpleasant (e.g., Monin, 2007), the urge to appease others might be so strong that it comes at the expense of less visible but potentially more effective responses. In other words, when fearing condemnation, people with overweight and obesity might feel urged to publicly demonstrate their moral motivation to change. These strategies, while potentially successful in managing the acute moral social-image threat, will often be less effective in the long run. On the other hand, when weight stigma focuses on incompetence as a less global flaw (de Hooge et al., 2010; Gausel and Leach, 2011), the self-critical experience of shame appears to stimulate a preference for slower and less visible responses to weight stigma, such as weight loss. This strategy is less visible to the social environment, but potentially more effective in long term. Thus, fear of being condemned by others seems to impair, while the self-critical experience of shame seems to facilitate, a slower but more efficient route to healthier living, and by such, self-change.

These findings align with earlier theorizing (e.g., Gausel and Leach, 2011; Gausel, 2013) and research on the concern for condemnation and defense strategies to minimize further condemnation or to escape current condemnation (e.g., Gausel et al., 2012, 2016, 2018). Our findings are consistent with the suggestion that perceived reparability of a shortcoming determines whether people respond more or less constructively (Leach and Cidam, 2015). Importantly, both responses should be considered functional with respect to their potential in managing the threat that results from being confronted with weight-stigma (e.g., de Hooge et al., 2010). While the experience of shame is unpleasant (e.g., Tangney and Dearing, 2002), it can be a motivator of positive change (Gausel and Leach, 2011) that may result in contemplation to change (Gausel and Brown, 2012; Lickel et al., 2014) and where relevant engage in constructive behavior (e.g., Gausel et al., 2012, 2016, 2018; Leach and Cidam, 2015).

Our second study was designed to address two main aims: First, we empirically test our reasoning that weight bias internalization predominantly reflects moral judgments, by considering BMI, weight stigma focusing on morality and competence, as well as concern for condemnation as antecedents of weight bias internalization in people across the weight spectrum. To the extent that weight bias internalization indeed reflects moral aspects of weight stigma, previous contributions suggest that it should be strongly associated with other-determined regulation of relevant behaviors such as dieting and exercising. By contrast, weight bias internalization should decrease self-determined regulation of relevant behaviors in the context of weight (Pelletier and Dion, 2007; Deci and Ryan, 2008).

## Study 2

### Materials and Methods

#### Participants and Design

Three-hundred-fifty-one U.S. American respondents were recruited using MTurk. Of those, three were excluded because their reported weight and height resulted in physically implausible BMI values (0.19, 3.87, and 11.08 kg/m^2^, respectively). The resulting sample of 348 respondents consisted of 181 females (52%) and 167 males (48%), *M*_Age_ = 37.15, *SD*_Age_ = 11.15, *M*_BMI_ = 26.78, *SD*_BMI_ = 6.78, range 15.34–65.10. The study was presented using the online survey tool Qualtrics^TM^ (see **Supplementary Appendix A** for the complete introduction). Prior to participating, respondents were informed that study participation was voluntary, that their individual responses would be completely anonymous and that filling in the questionnaire would take approximately 15 min. Based on this information, respondents were asked to provide informed consent before proceeding to the questionnaire. Respondents received $2 as compensation for their effort.

#### Measurements

**Supplementary Appendix A** provides an overview of all items assessed in this study. Besides the demographic variables reported above (age, sex, as well as weight and height to calculate BMI), the measures reflected three clusters of interest. First, we measured respondents’ perception of the public’s views on people with overweight and obesity as immoral and incompetent, as well as their concern for condemnation by others. Second, we measured the extent to which respondents had internalized weight bias using the Modified Weight Bias Internalization Scale (WBIS-M, Pearl and Puhl, 2014; α = 0.95) to gain more insights into the interplay between weight bias internalization and behavioral regulation. Third, to test our predictions concerning motivation more rigorously, we assessed respondents’ agreement with statements about their underlying motivation for dieting and exercising using scales that reflect the full spectrum from autonomous to controlled behavioral regulation (Ryan and Deci, 2000; Pelletier and Dion, 2007). Specifically, we assessed respondents’ agreement with statements about dieting (General Motivation Scale, GMS; Pelletier et al., 2004) and exercising (Behavioral Regulation in Exercise Questionnaire, BREQ-3; Markland and Tobin, 2004). Both instruments consist of six subscales reflecting SDT’s regulatory behavior along the continuum of self-determination (motivations for dieting: intrinsic α = 0.95, integrated α = 0.94, identified α = 0.86, introjected α = 0.80, external α = 0.91, and amotivation α = 0.94; motivations for exercising: intrinsic α = 0.97, integrated α = 0.93, identified α = 0.88, introjected α = 0.92, external α = 0.94, and amotivation α = 0.97). **Table 2** provides an overview of the means and standard deviations of all measurements.

**Table 2:** Means and standard deviations for Study 2.

	*M*	*SD*
Sex (1 = male, 2 = female)	1.48	0.50
Age	37.15	11.15
BMI	26.78	6.78
Public view immoral^a^	4.10	1.54
Public view incompetent^a^	4.79	1.52
Concern for condemnation^a^	3.16	1.97
WBIS-M^a^	3.17	1.54
Dieting^b^		
Intrinsic motivation	3.52	1.00
Integrated motivation	3.31	1.06
Identified motivation	4.02	0.72
Introjected motivation	2.97	0.96
External motivation	2.02	0.96
Amotivation	1.75	0.86
Exercising^b^		
Intrinsic motivation	2.90	1.21
Integrated motivation	3.05	1.13
Identified motivation	3.48	0.98
Introjected motivation	2.48	1.12
External motivation	1.78	0.91
Amotivation	1.59	0.86

*^*a*^Scale 1–7. ^*b*^Scale 1–5.*

### Results

#### Descriptive Analysis

**Table 3** provides an overview of the correlations between demographic variables, weight stigma focus, weight bias internalization, and motivation for dieting. **Table 4** provides the same correlations with respondents’ motivation for exercising. Below, using structural equation modeling, we test the prediction that weight bias internalization reflects moral aspects of weight stigma and is thus associated with less self-determined and more other-determined regulation of dieting and exercising.

**Table 3:** Correlations between demographic variables, BMI, weight stigma focus on morality and competence, concern for condemnation, weight bias internalization (WBIS-M), and types of motivation for dieting, Study 2.

	1	2	3	4	5	6	7	8	9	10	11	12
1. Sex^a^	1.00											
2. Age	–0.14*	1.00										
3. BMI	–0.01	0.00	1.00									
4. Public view immoral	–0.01	–0.20***	0.16**	1.00								
5. Public view incompetent	0.01	–0.23***	0.14**	0.67***	1.00							
6. Concern for condemnation	–0.06	–0.08	0.52***	0.27***	0.28***	1.00						
7. WBIS-M	–0.15**	–0.10	0.52***	0.29***	0.27***	0.83***	1.00					
8. Intrinsic motivation	–0.08	0.01	–0.13*	–0.06	–0.01	–0.17**	–0.20**	1.00				
9. Integrated motivation	–0.05	0.03	–0.28***	–0.04	–0.02	–0.30***	–0.32***	0.71***	1.00			
10. Identified motivation	–0.10	0.04	0.05	–0.04	0.05	–0.02	–0.05	0.55***	0.48***	1.00		
11. Introjected motivation	–0.11*	–0.15**	0.04	0.21***	0.21***	0.33***	0.41***	0.20***	0.16**	0.30***	1.00	
12. External motivation	0.07	–0.22***	0.19***	0.20***	0.06	0.29***	0.38***	–0.02	0.01	–0.05	0.32***	1.00
13. Amotivation	0.04	–0.15*	0.26***	0.28***	0.12*	0.44***	0.48***	–0.26***	–0.26***	–0.35***	0.14*	0.51***

*^∗^*p* < 0.05, ^∗∗^*p* < 0.01, ^∗∗∗^*p* < 0.001. ^*a*^1 = female, 2 = male.*

**Table 4:** Correlations between demographic variables, BMI, weight stigma focus on morality and competence, concern for condemnation, weight bias internalization (WBIS-M), and types of motivation for exercising, Study 2.

	1	2	3	4	5	6	7	8	9	10	11	12
1. Sex^a^	1.00											
2. Age	–0.14*	1.00										
3. BMI	–0.01	0.00	1.00									
4. Public view immoral	–0.01	–0.20***	0.16**	1.00								
5. Public view incompetent	0.00	–0.23***	0.14**	0.67***	1.00							
6. Concern for condemnation	–0.06	–0.08	0.52***	0.26***	0.28***	1.00						
7. WBIS-M	–0.15**	–0.10	0.52***	0.29***	0.27***	0.83***	1.00					
8. Intrinsic motivation	0.14**	–0.06	–0.20***	–0.02	–0.07	–0.28***	–0.28**	1.00				
9. Integrated motivation	0.17**	–0.07	–0.26***	–0.04	–0.03	–0.29***	–0.32***	0.78***	1.00			
10. Identified motivation	0.09	0.00	–0.23***	–0.03	0.01	–0.27	–0.30***	0.68***	0.80***	1.00		
11. Introjected motivation	–0.04	–0.13*	–0.01	0.15**	0.16**	0.18**	0.29***	0.20***	0.35**	0.42***	1.00	
12. External motivation	0.10	–0.22***	0.12*	0.25***	0.11*	0.26***	0.35***	–0.04	0.02	–0.05	0.27***	1.00
13. Amotivation	–0.05	–0.12*	0.10	0.22***	0.05	0.28***	0.30***	–0.24***	–0.27***	–0.43***	–0.09*	0.41***

*^∗^*p* < 0.05, ^∗∗^*p* < 0.01, ^∗∗∗^*p* < 0.001. ^a^1 = female, 2 = male.*

### Structural Regression Modeling

As in the first study, we specified the structural regression model using AMOS 23 with maximum-likelihood estimation. However, due to the manifold of the relations in this study, we specified two models; one for dieting and one for exercising.

#### Dieting Model

**FIGURE 2:**
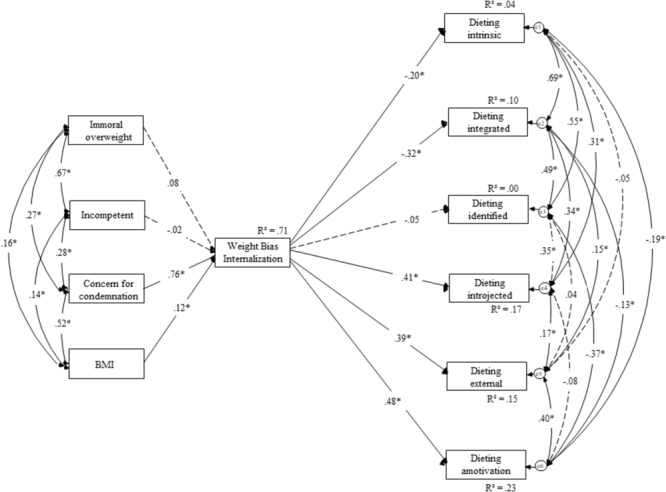
Structural equation model for the effects of weight stigma focusing on morality, competence, concern for condemnation, and BMI on weight bias internalization and dieting motivation for Study 2.

In the first model (**Figure 2**), we tested our hypothesis that through BMI, immorality, incompetence, and fear of condemnation (i.e., social-image concerns), weight bias internalization will negatively predict self-determined, autonomous regulation strategies (i.e., intrinsic, integrated, and identified motivation) and positively predict other-determined, controlled regulation strategies (i.e., introjected, amotivation, and external motivation). Even though the complexity of the model provided a significant chi-square, χ^2^(24) = 83.04, *p* < 0.001 (χ^2^/*df* = 3.46), our other fit indices indicated a good fit of the model (IFI = 0.965, CFI = 0.964, RMSEA = 0.084) according to Kline (2005) and MacCallum et al. (1996).

As expected, the concern for condemnation was a significant predictor of weight bias internalization (β = 0.76, *p* < 0.001), along with BMI (β = 0.12, *p* < 0.001) and the public view that people with overweight and obesity are immoral (β = 0.08, *p* = 0.032). Consistent with expectations, the public view that people with overweight and obesity are incompetent proved to be a non-significant predictor of weight bias internalization (β = -0.02, *p* = 0.60). These findings support our proposition that internalized weight bias reflects essentially moral concerns. Weight bias internalization was, as expected, a significant, negative predictor of intrinsic motivation (β = -0.20, *p* < 0.001) and of integrated motivation (β = -0.32, *p* < 0.001). However, it was unrelated to an identified motivation (β = -0.05, *p* = 0.33). In line with our hypotheses, weight bias internalization was a significant, positive predictor of introjected motivation (β = 0.41, *p* < 0.001), external motivation (β = 0.39, *p* < 0.001), and to amotivation (β = 0.48, *p* < 0.001).

#### Exercising Model

**FIGURE 3:**
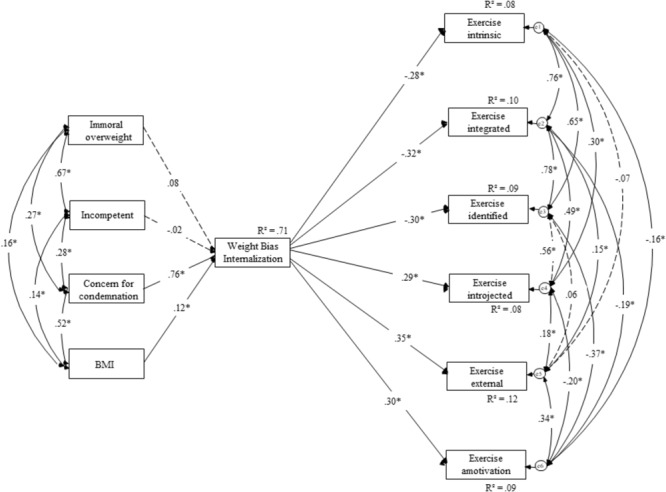
Structural equation model for the effects of weight stigma focusing on morality, competence, concern for condemnation, and BMI on weight bias internalization and exercising motivation for Study 2.

In the second model of Study 2 (**Figure 3**), we tested a similar model to the first, but this time exercise was the outcome variables. Again, our hypothesis was that weight bias internalization would negatively predict adaptive regulation strategies (i.e., intrinsic, integrated, and identified motivation) and positively predict maladaptive regulation strategies (i.e., introjected, amotivation, and external motivation). Despite a significant chi-square, χ^2^(24) = 62.57, *p* < 0.001 (χ^2^/*df* = 2.60), our main fit indices indicated a good fit of the model (IFI = 0.980, CFI = 0.980, RMSEA = 0.068) according to Kline (2005) and MacCallum et al. (1996).

Of course, the first part of our model was identical to the first model of Study 2. As expected, weight bias internalization was a significant, negative predictor of intrinsic motivation (β = -0.28, *p* < 0.001), integrated motivation (β = -0.32, *p* < 0.001), and to an identified motivation (β = -0.30, *p* < 0.001). In line with our hypotheses, weight bias internalization was a significant, positive predictor of introjected motivation (β = 0.29, *p* < 0.001), external motivation (β = 0.35, *p* < 0.001), and to amotivation (β = 0.30, *p* < 0.001).

### Discussion

Aligning with prior research (e.g., Latner et al., 2014), we found that people with overweight and obesity report more weight bias internalization. Study 2 advances our understanding of weight bias internalization by showing that it results from aspects of weight stigma that are related to morality, but not to competence, as well as concern for condemnation by others. In line with our hypothesis, concern for condemnation was a very strong and positive significant predictor of weight bias internalization. This means that people with overweight and obesity, who focus on the moral dimension of weight stigma and are concerned that others could condemn them, report the highest levels of weight bias internalization. As the model explains 71% of the variance in weight bias internalization, it suggests that these variables are the dominant reasons for weight bias internalization. Importantly, and consistent with our hypotheses, weight bias internalization was negatively associated with autonomous motivation and positively associated with controlled motivation. Thus, weight bias internalization impairs behavioral regulation that stems from intrinsic motivation and boosts behavioral regulation that is motivated by others’ judgments about the self (Ryan and Deci, 2000; Deci and Ryan, 2008). While this aligns with prior research showing that SDT is a viable theoretical framework for investigating motivation in people with overweight and obesity (e.g., Pelletier and Dion, 2007), it valuably advances prior research by identifying weight bias internalization as a crucial explanatory variable for motivation in people with overweight and obesity.

## General Discussion

The current research aimed to advance scholarly understanding of underlying mechanisms explaining more or less adaptive responses to weight stigma. To this end, we integrated different strands of so far unconnected research on SDT (Deci and Ryan, 2008), moral motivation (e.g., Täuber and van Zomeren, 2012, 2013; Täuber et al., 2015), and shame (e.g., Gausel and Leach, 2011). Study 1 demonstrated that when people with overweight and obesity are confronted with weight stigma suggesting they are immoral and thus globally flawed (de Hooge et al., 2010; Gausel and Leach, 2011), they report increased fear of condemnation (i.e., their social-image). Fear of condemnation was associated with a preference for quickly implementable, highly visible responses to weight stigma. We have suggested that the observed preference for such responses reflects a functional approach to managing acute threats to moral image. Thus, fear of condemnation does not appear to be beneficial in supporting people with overweight and obesity to change their body weight. This finding is in alignment with previous research (e.g., Vartanian and Novak, 2011; Jackson et al., 2015), that experiences of weight stigma lead to maladaptive responses. On the other hand, Study 1 demonstrated that when people with overweight and obesity are confronted with weight stigma suggesting they are incompetent and thus less globally flawed (de Hooge et al., 2010; Gausel and Leach, 2011), they experience shame. Shame motivated a slower, less visible, but probably more efficient route to healthier living, and by such, self-change. This finding supports earlier theorizing (Gausel and Leach, 2011) and empirical research (Gausel et al., 2012, 2016, 2018; Lickel et al., 2014) that felt shame is an unpleasant, yet positive predictor of constructive motivation and self-change. Our findings also align a recent meta-analysis on the association of shame with constructive responses (Leach and Cidam, 2015), which demonstrated that the crucial factor determining whether people want to improve vs. defend the self after failure is the extent to which the failure is seen as repairable. Study 1 findings thus align with our notion that weight stigma is perceived as less repairable when it revolves around immorality compared to incompetence. Therefore, weight stigma that emphasizes that people with overweight and obesity are immoral elicits fear of condemnation and will lead to preferences for responses that allow to quickly and visibly show others that one is willing to improve and change behavior (Gausel and Leach, 2011; Gausel, 2013). These responses, we suggest, are functional to manage an acutely threatened moral social image, but potentially less effective in achieving long-term successes in healthier eating and living. Given that the discourse about obesity is highly moralized (e.g., Townend, 2009), Study 1 findings therefore strengthen the argument that weight stigma is not beneficial in supporting people with overweight and obesity to change their body weight. By contrast, our findings highlight that there is a need to change the discourse relating to overweight and obesity as seen in public policy, media, and heath campaigns to reduce feelings of condemnation.

Study 2 findings extend our understanding of weight bias internalization and the reasons it is associated with maladaptive responses. Our findings demonstrate that weight bias internalization results from moral, but not competent, aspects of weight stigma, as well as concern for condemnation by others. Concern for condemnation, reported by people with overweight and obesity, was a very strong predictor of weight bias internalization. Thus, weight bias internalization appears to reflect a view of the self as immoral. As we have outlined in our theoretical rationale, threats to morality are experienced as very averse and will likely promote responses that are quick to perform and visible to the social environment, but potentially less effective to lose weight in the long-run. Our findings also extend recent research (Romano et al., 2018) reporting that weight stigma leads to increased food intake because it poses a threat to social identity. We offer an important qualifier of these findings by showing that not any threat, but moral threats in particular, will likely lead to maladaptive responses to weight stigma.

Further, because weight bias internalization appears to reflect an internalized image of oneself as immoral and thus as globally flawed (de Hooge et al., 2010; Gausel and Leach, 2011), people who have internalized weight bias are likely to experience a constant state of acute threat to their moral social image, thereby focusing on behaviors that demonstrate to their social environment that they are indeed moral people. Unfortunately, these behaviors are likely to be less efficient in losing weight or eating healthier. Indeed, Study 2 provided strong support for the proposition that weight bias internalization promotes less self-determined and more other-determined regulation of dieting and exercising. Ample research has demonstrated that other-determined behavioral regulation is associated with lower psychological functioning and well-being (e.g., Ryan and Deci, 2000; Pelletier and Dion, 2007). Thus, our findings might valuable advance scholarly understanding of why weight bias internalization is related to medical comorbidities, greater impairment in the physical and mental domains of life (Latner et al., 2013, 2014), as well as to variance in eating disorder psychopathology (Durso et al., 2012).

### Strengths, Limitations, and Future Research

By integrating so far unconnected lines of research, we have derived innovative predictions concerning the mechanisms underlying responses to weight stigma among people with overweight and obesity. We have further advanced insights into weight bias internalization, showing that it is essentially a moral threat, thereby shedding light on the motivational consequences of weight bias internalization. The two studies complement each other in their methods (experimental and survey approach), in their focus on people with overweight and obesity (Study 1), and on the complete weight spectrum (Study 2). Both studies offer valuable insights into mechanisms underlying maladaptive and adaptive responses to weight stigma. Study 2 further provides valuable insights into the etiology of weight bias internalization, pointing out the relevance of moral construal and social image concerns.

Due to the study design, to collect information about participants’ weight status, they self-reported their height and weight so that we could establish their BMI. Body mass is typically prone to underreporting and therefore might be inaccurate. Another potential limitation, in line with previous research (Rothman, 2008), is the use of BMI as an indicator of overweight and obesity. Another study design might have allowed for more direct and thus, accurate measures of body fatness to have been used. Future research should tease out the findings of the current research through real-world application of competence rather than morality-based discourse. Research should examine the impact on behavior change to explore whether the findings of the current study are translatable to, for instance, supporting public health campaign engagement and public response to media discourse (e.g., potentially reduced internalization of weight bias).

Relatedly, future research should investigate factors that might protect people from the negative effects of weight-stigma. Such factors concern, for instance, cultural differences and subjective perceptions of weight. Specifically, overweight is not considered negative in all countries and cultures (Hebl and Heatherton, 1998; Padgett and Biro, 2003), which should affect whether weight is moralized, but might also affect how people with overweight and obesity respond to moral weight-stigma. Likewise, Major et al. (2014) showed that people feel less threatened by weight-stigmatizing messages when they don’t perceive themselves to be overweight – even when they are objectively overweight. This research suggests that there are factors besides objective weight that affect how people respond to weight-stigma, which have not been considered in the present research.

### Practical Implications

Our findings highlight the potential implications of weight bias internalization, where discourse that informs that overweight and obesity is immoral – as discussed in previous literature (e.g., Crandall, 1994; Crandall and Schiffhauer, 1998; Crandall et al., 2001; Hoverd and Sibley, 2007) – appears to be an influential factor in why people internalize weight bias. Our research indicates that to reduce weight bias internalization and potentially the associated impacts of weight bias internalization (e.g., anxiety and depression), suggestions that overweight and obesity are immoral needs to be removed. Importantly, while our research offers strong pointers toward replacing the moral construal of weight by an emphasis on competence as a strategy to avoid maladaptive behaviors, we wish to nuance this conclusion. We have examined the motivational relevance of different aspects of weight stigma, revolving around incompetence and immorality, respectively. To suggest that strategies aiming to motivate weight loss and healthier eating should emphasize incompetence rather than immorality is based on our findings, but simply means picking the lesser of two evils associated with a stigmatized discourse about weight.

Indeed, we strongly encourage the counter-moralization of weight-related discourse and campaigning, rather than substituting suggestions of immorality with suggestions of incompetence. In contrast to moralizing information about weight, counter-moralizing information has been shown to motivate people with obesity to snack more healthily (Mulder et al., 2015). This aligns with findings by Täuber and van Zomeren (2012) showing that in comparison to moralization, morally neutral information elicited greater motivation for change after a shortcoming. We suggest that the public, governments, and the media elicit morally neutral, non-threatening beliefs about overweight and obesity, as these appear to facilitate behavioral regulation bolstering psychological functioning. It is our suggestion that the widespread moral discourse about health and weight should raise red flags among politicians, doctors, and the broader public and its implications for behavioral regulation in people with overweight and obesity. It appears that such discourse, rather than being motivating, will lead to vigilance for moral condemnation and social exclusion in people with overweight and obesity, thereby resulting in maladaptive behavior. We consequently call for more research on interventions targeting communication by the public, politicians, and institutions, that will prevent maladaptive responses to weight stigma reported in the present research.

## Conclusion

The two studies presented above provide innovative insights concerning strategies to bolster resilience and psychological functioning of people with overweight and obesity, and they offer a strong pointer to public’s responsibilities to use unbiased, morally neutral language. The current studies have novel findings that highlight the impact of concerns of condemnation and influence of presenting overweight and obesity as immoral. Our findings provide further evidence of the detrimental impact of exposure to stigmatizing and discriminatory portrayal of weight stigma and offer valuable insights into the moral core and thus motivational relevance of weight bias internalization. Given the impact of internalized weight bias on physical and mental health outcomes and maladaptive behavioral responses, the current research holds strong implications for the design and communication of public health policy and campaigns, healthcare, and media portrayal. The complexity of obesity as evidenced in the Foresight Report (Butland et al., 2007) demonstrates the vast array of contributing factors, many of which are outside of an individual’s control. This highlights the inaccuracy of presenting overweight and obesity as immoral. Coupled with the current research findings that demonstrate perceptions of overweight and obesity as immoral is a key contributor to internalized weight bias and extent literature that internalized weight bias leads to health decrements and maladaptive coping, we call for an end to debates about the morality of overweight and obesity. Our research underscores the need to change the narrative and discourse relating to obesity. Moral debates about overweight and obesity should be replaced with a focus on supporting positive health behaviors through morally neutral language.

## Ethics Statement

This study was carried out in accordance with the recommendations of the Ethical Commission of the Behavioral Research Lab of the Faculty of Economics and Business (University of Groningen) with written informed consent from all subjects. All subjects gave written informed consent in accordance with the Declaration of Helsinki. The protocol was approved by the Ethical Commission of the Behavioral Research Lab.

## Author Contributions

ST contributed to the conception and design of Study 1, and wrote the first draft of the manuscript. NG performed the statistical analyses of Studies 1 and 2. NG and SF wrote sections of the manuscript. All authors conceived and designed Study 2, and contributed to manuscript revision, and read and approved the submitted version.

## Conflict of Interest Statement

The authors declare that the research was conducted in the absence of any commercial or financial relationships that could be construed as a potential conflict of interest.

## Footnotes

1 Note that the authors are by no means suggesting that shaming people with overweight and obesity is legitimate. Our starting point is the observation that weight stigma predominantly revolves around notions of incompetence and immorality, and we aim to examine the, as we predict, different motivational responses to these aspects of stigma.
