# Supplementary material for: Weight Bias Internalization: The Maladaptive Effects of Moral Condemnation on Intrinsic Motivation
Source: Front Psychol. 2018 Sep 27;9:1836. doi: 10.3389/fpsyg.2018.01836 (PMC6170635; doi:10.3389/fpsyg.2018.01836)
Supplement: Supplementary file 1 [file Table_1.DOCX]

**Appendix A**

**Manipulations Study 1**

The following text summarizes a recent news article about research results on health costs, health and lifestyles. Please read the text carefully. Later, questions about this text will follow.

Lifestyle increasingly harmful?

www.nu.nl/gezondheid d.d. 02-10-2016

In the last decades there has been a crisis concerning the welfare state in most Western European countries. Health care costs continue to rise and it is still unclear how these can be paid in the future. In The Netherlands this is often a point of discussion.

More and more information is available about the relationship between health and lifestyle. Several researchers have stressed that a person's health is partly determined by the way of life; for example how much someone eats, drinks and smokes. As a result, an unhealthy lifestyle can lead to increased healthcare costs. Public opinion is the last years that people with an unhealthy lifestyle, and therefore also people with an unhealthy weight, are **immoral**/ **incompetent**.

**Measures Study 1**

*Demographics*

How tall are you? How much do you weigh? What is your sex? How old are you?

*Public view of overweight people*

(from 1 = strongly disagree to 5= strongly agree)

Public opinion is that people with an unhealthy weight are immoral.

Public opinion is that people with an unhealthy lifestyle are immoral.

Public opinion is that people with an unhealthy weight are incompetent.

Public opinion is that people with an unhealthy lifestyle are inadequate.

*Fear of condemnation*

Others might not have the same respect for me because of my weight.

I can be rejected by others because of my weight.

I think I can be isolated from others because of my weight.

*Shame*

I feel disgraced thinking about my weight.

I feel ashamed thinking about my weight.

I feel humiliated thinking about my weight.

*Motivation to seek information*

I am going to find information about healthy living.

I am going to motivate my family and friends to live healthier.

*Motivation to lose weight*

I would like to lose weight.

I would like to have a healthy weight.

**Introduction Study 2**

Thank you for participating in our study! We are interested in how people think about weight, and how they manage with their own and other’s weight. We appreciate your efforts in filling in this survey. We intend to use the findings of this survey to inform policy makers, scholars, and the public who are interested in weight. The survey has three main parts: the first asks about your personal background and weight-related attitudes; the second focuses on how you think about your own and others weight; and the third part focuses on your weight-related motivation and behaviours.

**Measures Study 2**

*Demographics*

How tall are you? How much do you weigh? What is your sex? How old are you?

*Construal of weight in moral vs. non-moral terms*

(from 1 = strongly disagree to 7= strongly agree)

Public opinion views higher weight people as incompetent.

Public opinion views higher weight people as immoral.

*Concern of condemnation*

(from 1 = not at all to 7 = very much)

Others might not have the same respect for me because of my weight.

I can be rejected by others because of my weight.

I think I can be isolated from others because of my weight.

*Modified Weight Bias Internalization Scale (WBIS-M)*

(from 1 = strongly disagree to 7= strongly agree)

Because of my weight, I feel that I am just as competent as anyone. (reverse)

I am less attractive than most other people because of my weight.

I feel anxious about my weight because of what people might think of me.

I wish I could drastically change my weight.

Whenever I think a lot about my weight, I feel depressed.

I hate myself for my weight.

My weight is a major way that I judge my value as a person.

I don’t feel that I deserve to have a really fulfilling social life, because of my weight.

I am OK being the weight that I am. (reverse)

Because of my weight, I don’t feel like my true self.

Because of my weight, I don’t understand how anyone attractive would want to date me.

*Motivation: Eating regulation (Pelletier et al., 2004)*

(1 = not at all true, 2 = not true, 3 = moderately true, 4 = true, 5 = completely true)

Intrinsic motivation

It is fun to create meals that are good for my health.

I like to find new ways to create meals that are good for my health.

I take pleasure in fixing healthy meals.

For the satisfaction of eating healthy.

Integrated regulation

Eating healthy is an integral part of my life.

Eating healthy is part of the way I have chosen to live my life.

Regulating my eating behaviors has become an integral part of who I am.

Eating healthy is congruent with other important aspects of my life.

Identified regulation

I believe it will eventually allow me to feel better.

I believe it’s a good thing I can do feel better about myself in general.

It is a good idea to try to regulate my eating behaviors.

It is a way to ensure long-term health benefits.

Introjected regulation

I don’t want to be ashamed of how I look.

I feel I must absolutely be thin.

I would feel ashamed of myself if I was not eating healthy.

I would be humiliated of I was not in control of my eating behaviors.

External regulation

Other people close to me insist that I do.

Other people close to me will be upset if I don’t.

People around me nag me to do it.

It is expected of me.

Amotivation

I don’t really know. I truly have the impression that I’m wasting my time trying to regulate my eating behaviors.

I don’t know why I bother.

I can’t really see what I’m getting out of it.

I don’t know. I can’t see how my efforts to eat healthy are helping my health situation.

*Motivation: Exercising (BREQ-3) (Markland & Tobin, 2004)*

(1 = not at all true, 2 = not true, 3 = moderately true, 4 = true, 5 = completely true)

Intrinsic motivation

I exercise because it’s fun.

I enjoy my exercise sessions.

I find exercise a pleasurable activity.

I get pleasure and satisfaction from participating in exercise.

Integrated regulation

I exercise because it is consistent with my life goals.

I consider exercise part of my identity.

I consider exercise a fundamental part of who I am.

I consider exercise consistent with my values.

Identified regulation

It’s important to me to exercise regularly.

I value the benefits of exercise.

I think it is important to make the effort to exercise regularly.

I get restless if I don’t exercise regularly.

Introjected regulation

I feel guilty when I don’t exercise.

I feel ashamed when I miss an exercise session.

I feel like a failure when I haven’t exercised in a while.

I would feel bad about myself if I was not making time to exercise.

External regulation

I exercise because other people say I should.

I take part in exercise because my friends/family/partner say I should.

I exercise because others will not be pleased with me if I don’t.

I feel under pressure from my friends/family to exercise.

Amotivation

I don’t see why I should have to exercise.

I can’t see why I should bother exercising.

I don’t see the point in exercising.

I think exercising is a waste of time.
